# Participation and performance trends in elderly marathoners in four of the world’s largest marathons during 2004–2011

**DOI:** 10.1186/s40064-015-1254-6

**Published:** 2015-08-29

**Authors:** Baschir Ahmadyar, Christoph Alexander Rüst, Thomas Rosemann, Beat Knechtle

**Affiliations:** Institute of Primary Care, University of Zurich, Zurich, Switzerland; Facharzt FMH für Allgemeinmedizin, Gesundheitszentrum St. Gallen, Vadianstrasse 26, 9001 St. Gallen, Switzerland

**Keywords:** Master athlete, Running, Age group, Gender

## Abstract

Performance and age of elite marathoners is well known. Participation and performance trends of elderly marathoners (75 years and older) are not well investigated. This study investigated participation and performance trends in elderly marathoners older than 75 years competing during 2004–2011 in four races (Berlin, New York, Chicago and Boston) of the ‘World Marathon Majors’ using mixed-effects regression models. Participation for women and men remained unchanged at 17 and 114, respectively, during the investigated period. For all finishers, marathon race times showed a significant and positive trend for gender, calendar year and age. For the annual fastest, calendar year and age showed a significant and positive trend. For the annual three fastest, gender, calendar year and age showed a significant and positive trend. The gender difference for the annual fastest and the annual three fastest showed no change across years. For the annual fastest and the annual three fastest, race times were fastest in the youngest age group (75–79 years) and slowest in the oldest age group (85–89 and 80–84 years, respectively). The gender difference in marathon race times remained unchanged across years at 19.7 ± 11.2, 28.1 ± 23.8 and 41.9 ± 22.6 % for the annual fastest in age groups 75–79, 80–84 and 85–89 years, respectively. For the annual three fastest men and women in age groups 75–79 and 80–84 years, the values were 23.7 ± 3.2 and 30.0 ± 13.2 %, respectively. In summary, for marathoners older than 75 years participating during 2004–2011 in four of the largest marathons in the world, participation for female and male runners remained unchanged, the fastest women and men became slower across years and the gender difference in performance remained unchanged. These findings might be the results of the relatively short period of time of 8 years. Future studies might investigate the performance trends in a large city marathon across a longer period of time.

## Background

The first modern marathon run was held in the 1908 Olympic Games in London (Wilcock 2008) where Johnny Hayes won the race in 2:55:18 h:min:s (Predel 2014). The current world records for men are 2:02:57 h:min:s (Dennis Kimetto 2014, http://www.iaaf.org/athletes/kenya/dennis-kipruto-kimetto-265313) and 2:15:25 h:min:s (Paula Radcliffe 2003, http://www.iaaf.org/athletes/great-britain-ni/paula-radcliffe-62914) for women. With the best age to achieve marathon records is ~31–32 years (Hunter et al. 2011; Berthelot et al. 2012), However, marathons are not the exclusive domain of the elite, with the vast majority of marathon participants being non-elites and spanning a wide range of ages. As illustrated by: (1) in the ‘New York City Marathon’, the numbers of male and female finishers increased between the decades 1980–1989 and 2000–2009 from 170,523 to 350,919 (Lepers and Cattagni 2012); (2) since 1976, the number of finishers older than 40 years has constantly increased (Burfoot 2007); and (3) runners older than 70 years accounted for ~0.6 % in the ‘New York City Marathon’ during the 2000–2009 period (Lepers and Cattagni 2012).

Studies investigating participation and performance trends of age group marathoners (Jokl et al. 2004; Leyk et al. 2007; Lepers and Cattagni 2012) have shown significant improvements in running times that in the last 30 years (Lepers and Cattagni 2012). A recent study reported significant improvements in marathon race times in men older than 64 years and women older than 44 years competing in the ‘New York City Marathon’ (Lepers and Cattagni 2012). Whilst these results may not be representative because only the data of the ‘New York City Marathon was used in the analysis, it might be suggested that both male and female age group runners have not yet reached their limits in marathon running. To verify these indications and overcome potential selection biases, the study presented here has incorporated data from multiple marathon races.

Additionally, recent studies investigating performances of age group marathoners (Jokl et al. 2004; Leyk et al. 2007; Lepers and Cattagni 2012) have failed to include elderly runners such as geriatric athletes (Jackson and Hynninen 1994) older than 75 years. This study has therefore examined the hypothesis of both an increase in participation and an improvement in performance in elderly marathoners (>75 years) by investigating marathon race times achieved in four of the largest city marathons as part of the ‘World Marathon Majors’ held between 2004 and 2011.

## Methods

### Ethics

The study was approved by the Institutional Review Board of St. Gallen, Switzerland, with a waiver of the requirement for informed consent given that the study involved the analysis of publicly available data.

### Data sampling and data analysis

Marathon race times of female and male finishers in four of the world’s largest city marathons were collected such as the ‘BMW Berlin Marathon’, the ‘ING New York City Marathon’, the ‘BOA Chicago Marathon’ and the ‘Boston Marathon’. These four races are part of the ‘World Marathon Majors’ with six races held in the cities of Tokyo, Boston, London, Berlin, Chicago, and New York (www.worldmarathonmajors.com). For the present intention to study age group athletes older than 75 years, we were not able to include the races held in London (www.virginmoneylondonmarathon.com/en-gb) and Tokyo (www.tokyo42195.org) since age group runners in London are only recorded as runners older than 70 years with no further separation in older age groups and no age groups are recorded in the Tokyo Marathon. Therefore, race results were obtained from the race websites ‘BMW Berlin Marathon’ (www.bmw-berlin-marathon.com), ‘ING New York City Marathon’ (www.tcsnycmarathon.org), ‘BOA Chicago Marathon’ (www.chicagomarathon.com) and ‘Boston Marathon’ (www.baa.org). Since the time frames of the four races were different, we restricted our data analysis to the time period 2004–2011 where full data from all four races were available.

### Statistical analysis

A potential change in participation across years was investigated using regression analysis. Gender difference (GD) was calculated using the equation [(time for women) – (time for men)/(time for men) × 100]. A mixed-effects regression model with finisher as random variable to include finishers who completed several races was used to calculate changes in performance of successful finishers. We included gender, age, squared age (i.e. because performance increases at decreasing rate with age) and calendar year as fixed variables. We also considered interaction effects between gender and age. The final model was selected by means of Akaike information criterion (AIC). Analysis of variance (ANOVA) was used to investigate differences in performance between the annual and the annual three fastest of female and male age groups. Statistical analyses were performed using IBM SPSS Statistics (Version 22, IBM SPSS, Chicago, IL, USA) and GraphPad Prism (Version 6.01, GraphPad Software, La Jolla, CA, USA). Significance was accepted *p* < 0.05. Data in the text and figures are given as mean ± standard deviation (SD).

## Results

### Participation trends

**Fig. 1:**
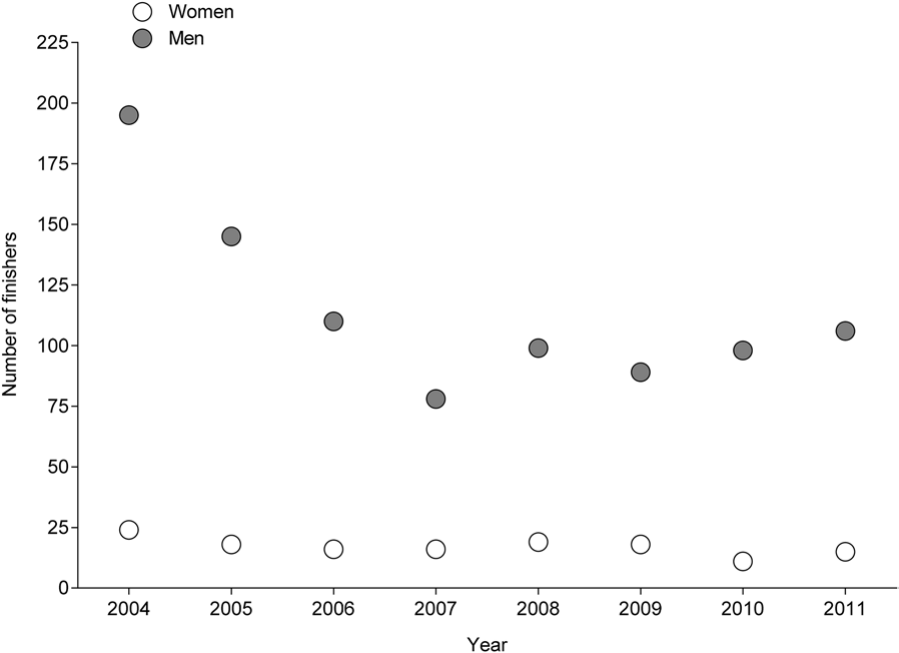
Participation of female and male finishers aged >75 years in all four races from 2004 to 2011

**Fig. 2:**
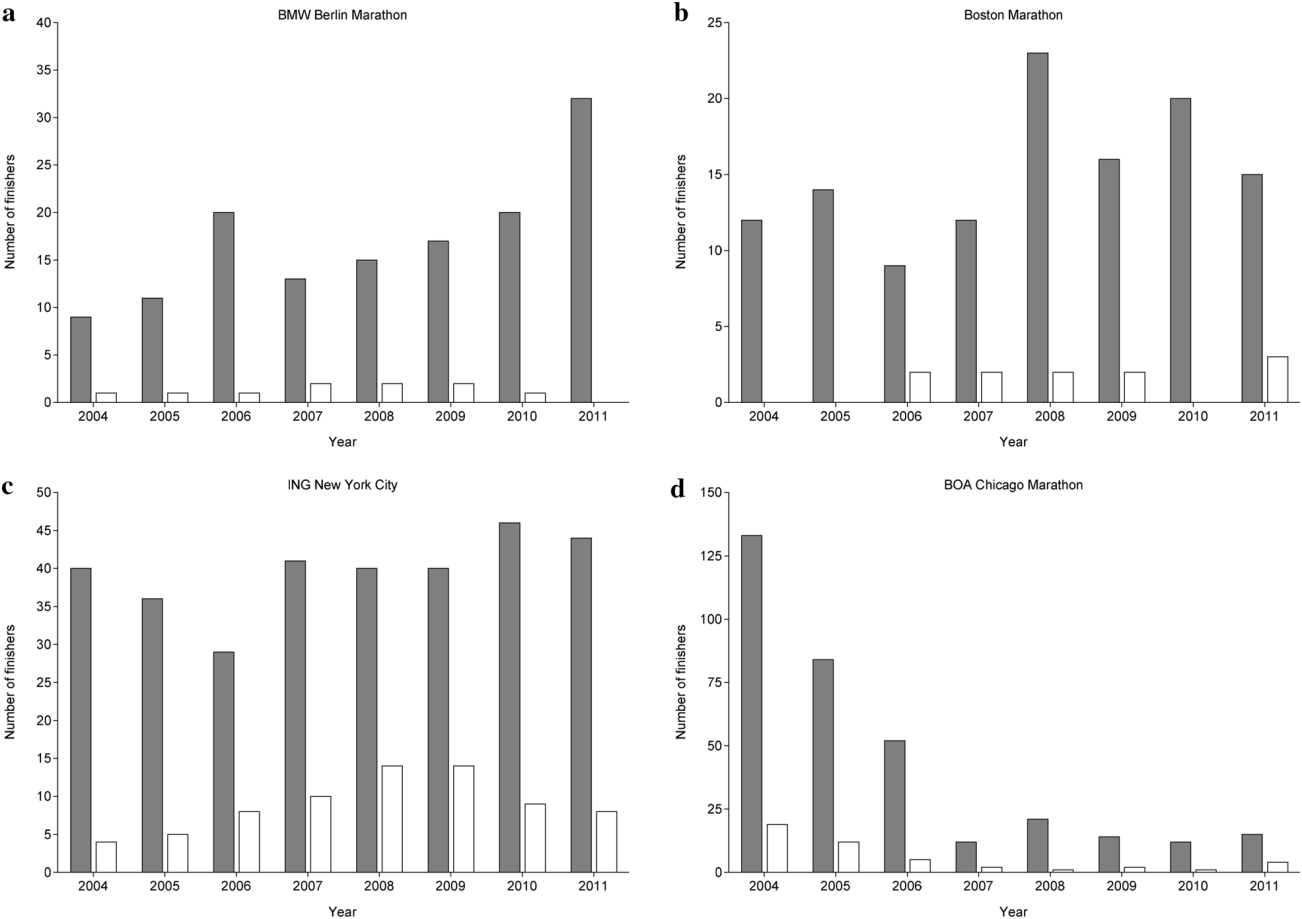
Number of female (*white bars*) and male (*grey bars*) finishers aged > 75 years for ‘BMW Berlin Marathon’ (**a**), ‘Boston Marathon’ (**b**), ‘ING New York City Marathon’ (**c**) and ‘BOA Chicago Marathon’ (**d**)

**Fig. 3:**
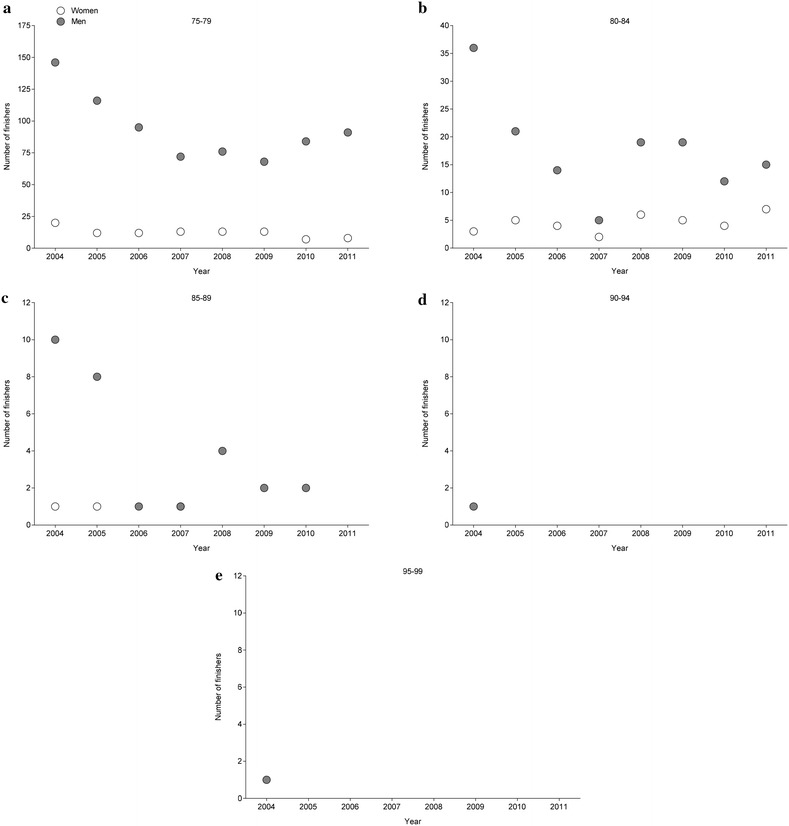
Participation of female and male finishers in all four races in age groups 75–79 years (**a**), 80–84 years (**b**), 85–89 years (**c**), 90–94 years (**d**) and 95–99 years (**e**)

Between 2004 and 2011, the number of finishers aged > 75 years remained unchanged for women (r^2^ = 0.39, *p* = 0.095) and men (r^2^ = 0.48, *p* = 0.055) at 17 and 114, respectively (Fig. 1). Most of the finishers competed in the ‘ING New York City Marathon’ (Fig. 2) and most of the finishers were ranked in age group 75–79 years (Fig. 3). In the ‘BMW Berlin Marathon’, the number of female finishers remained unchanged (r^2^ = 0.03, *p* = 0.696) whereas the number of male finishers increased (r^2^ = 0.65, *p* = 0.015). In the ‘Boston Marathon’, the number of female (r^2^ = 0.27, *p* = 0.190) and male (r^2^ = 0.28, *p* = 0.174) finishers remained unchanged. In the ‘ING New York City Marathon’, the number of female (r^2^ = 0.31, *p* = 0.151) and male (r^2^ = 0.38, *p* = 0.101) finishers remained unchanged. In the ‘BOA Chicago Marathon’, however, the number of female (r^2^ = 0.59, *p* = 0.026) and male (r^2^ = 0.65, *p* = 0.008) finishers decreased.

### Performance trends and gender differences

**Fig. 4:**
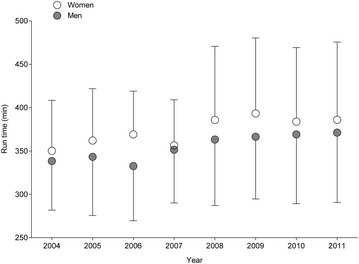
Marathon race times for all female and male finishers aged > 75 years in all four races

**Fig. 5:**
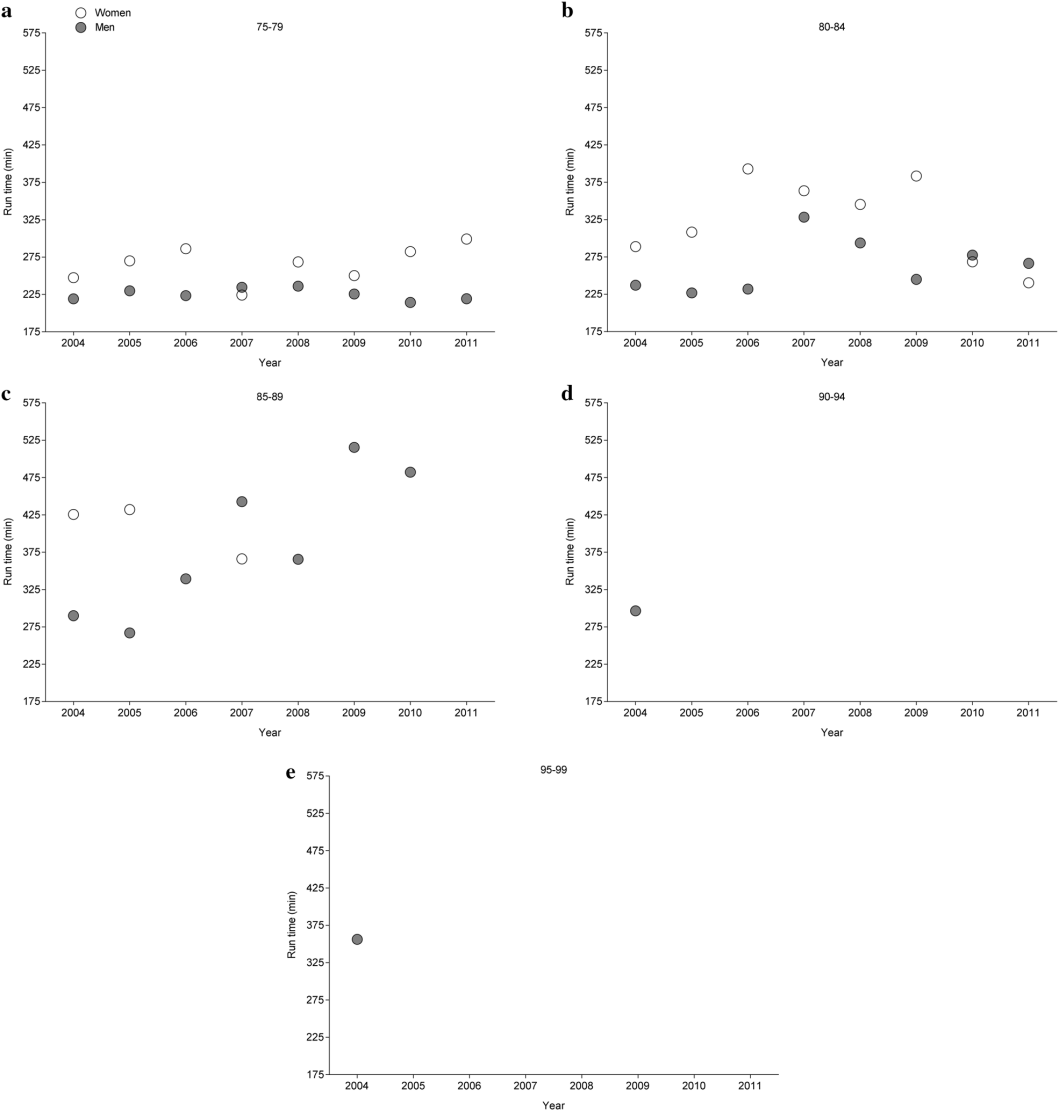
Marathon race times of the annual fastest female and male finishers in all four races in age groups 75–79 years (**a**), 80–84 years (**b**), 85–89 years (**c**), 90–94 years (**d**) and 95–99 years (**e**)

**Fig. 6:**
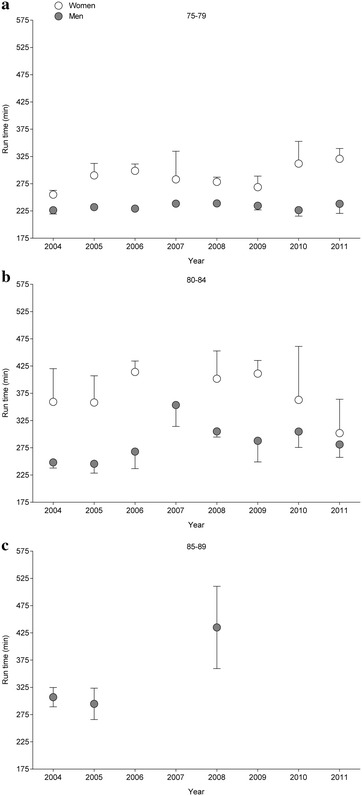
Marathon race times of the annual three fastest female and male finishers in all four races in age groups 75–79 years (**a**), 80–84 years (**b**), and 85–89 years (**c**)

Marathon race times for all finishers (Fig. 4) showed a significant and positive trend for gender, calendar year and age (Table 1). Considering the annual fastest (Fig. 5), calendar year and age showed a significant and positive trend (Table 1). For the annual three fastest (Fig. 6), gender, calendar year and age showed a significant and positive trend (Table 1). Significant interactions were found in all finishers for gender and age.

**Table 1:** Results of mixed effects regression analyses for race time for all, the annual fastest and the annual three fastest finishers

Parameter	Estimate	SE	*df*	t	*p* value
*All finishers*					
Constant term	−17,025.92	1334.69	921.87	−12.75	<0.0001
(Gender = f)	23.34	8.10	775.06	2.87	0.004
Calendar year	8.65	0.66	922.02	13.01	<0.0001
Cage	5.21	1.06	808.90	4.91	<0.0001
Cage^2^	−0.12	0.09	595.08	−1.32	0.187
(Gender = f) × cage	−7.43	3.83	787.02	−1.93	0.053
(Gender = f) × cage^2^	1.22	0.53	773.14	2.31	0.021
*Annual fastest*					
Constant term	−10,780.25	5967.83	41.83	−1.80	0.078
(Gender = f)	29.01	22.49	42.56	1.28	0.204
Calendar year	5.48	2.97	41.81	1.84	0.072
Cage	9.57	11.76	31.11	0.81	0.422
Cage^2^	−1.22	0.68	22.00	−1.78	0.088
(Gender = f) × cage	0.53	15.83	30.26	0.03	0.973
(Gender = f) × cage^2^	0.83	1.65	39.67	0.50	0.617
(Gender = f) × (age group = 85–89)	97.22	97.18	43.08	1.00	0.323
(Gender = f) × (age group = 80–84)	27.35	39.34	43.96	0.69	0.491
(Gender = m) × (age group = 95–99)	481.64	182.05	15.84	2.64	0.018
(Gender = m) × (age group = 90–94)	234.69	102.33	19.55	2.29	0.033
(Gender = m) × (age group = 85–89)	209.87	86.63	25.08	2.42	0.023
(Gender = m) × (age group = 80–84)	15.55	45.97	17.64	0.33	0.739
*Annual three fastest*					
Constant term	−8462.10	3487.14	98.90	−2.42	0.017
(Gender = f)	−107.05	47.30	60.97	−2.26	0.027
Calendar year	4.42	1.73	98.83	2.55	0.012
Cage	11.21	5.17	53.16	2.16	0.035
Cage^2^	−1.69	0.41	36.57	−4.05	<0.0001
(Gender = f) × cage	0.27	7.20	47.77	0.03	0.969
(Gender = f) × cage^2^	1.55	0.87	58.95	1.76	0.082
(Gender = f) × (age group = 75–79)	−11.01	17.29	36.53	−0.637	0.528
(Gender = m) × (age group = 75–79)	−181.52	42.53	55.53	−4.26	<0.0001
(Gender = m) × (age group = 80–84)	−149.15	31.73	69.43	−4.70	<0.0001

*f* female, *m* male, *cage* centered age, *SE* standard error

For the annual fastest and the annual three fastest, race times were fastest in the youngest age group (75–79 years) and slowest in the oldest age group (85–89 and 80–84 years, respectively). For the annual fastest women, race times were 4:25 ± 0:24, 5:20 ± 0:55 and 6:47 ± 0.36 h:min for age groups 75–79, 80–84 and 85–89 years, respectively (*p* = 0.029). For the annual fastest men (*p* = 0.0021), race times were 3:44 ± 0:07, 4:23 ± 0:35, and 6:25 ± 1:35 h:min, respectively. For the annual three fastest, women in age group 75–79 years (4:47 ± 0:21 h:min) were faster than women in age group 80–84 years (6:12 ± 0:40 h:min) (*p* = 0.031). Similarly, the annual three fastest men in age group 75–79 years (4:05 ± 0:46 h:min) were faster than the annual three fastest men in age group 80–84 years (4:46 ± 0:35 h:min) (*p* = 0.0078).

The GD for the annual fastest and the annual three fastest showed no change (Table 2). The GD in marathon race times remained unchanged across years at 19.7 ± 11.2, 28.1 ± 23.8 and 41.9 ± 22.6 % for the annual fastest in age groups 75–79, 80–84 and 85–89 years, respectively (Table 3). For the annual three fastest men and women in age groups 75–79 and 80–84 years, the values were 23.7 ± 3.2 and 30.0 ± 13.2 %, respectively (Table 4).

**Table 2:** Results of mixed effects regression analyses for gender difference in race time for the annual fastest and the annual three fastest finishers

Parameter	Estimate	SE	*df*	t	*p* value
*Annual fastest finishers*					
Constant term	2749.73	3812.86	19.000	0.721	0.480
Calendar year	−1.36	1.89	19.000	−0.716	0.483
Cage gender difference	4.07	6.60	19.000	0.617	0.545
Cage^2^ gender difference	0.17	0.87	19.000	0.195	0.848
(Gender = f) × (age group = 85–89)	−42.64	82.73	19.000	−0.515	0.612
(Gender = f) × (age group = 80–84)	−14.66	27.14	19.000	−0.540	0.595
*Annual three fastest finishers*					
Constant term	2202.04	1829.08	35.12	1.20	0.237
Calendar year	−1.08	0.91	35.05	−1.18	0.243
Cage gender difference	−2.01	3.05	44.78	−0.65	0.514
Cage^2^ gender difference	0.57	0.51	44.92	1.11	0.272
(Gender = f) × (age group = 75–79)	−7.27	12.85	44.79	−0.56	0.574

*f* female, *cage* centered age, *SE* standard error

**Table 3:** Race times (h:min) for the annual fastest women and men in age groups 75–79, 80–84 and 85–89 years with GD

Years	75–79 years			80–84 years			85–89 years		
	Women	Men	GD	Women	Men	GD	Women	Men	GD
2004	4:07	3:39	0:28	4:48	4:57	0:09	7:05	4:50	02:15
2005	4:29	3:49	0:40	4:28	4:46	0:18	7:12	4:37	02:45
2006	4:48	3:43	1:05	6:32	3:52	3:20		5:39	
2007	3:54	3:54	0:00	6:03	5:28	0:35	6:06	7:22	01:16
2008	4:28	3:56	0:32	5:45	4:53	0:52		6:05	
2009	4:10	3:45	0:25	6:23	4:05	2:18		8:35	
2010	4:42	3:34	1:08	4:28	4:37	0:09		8:02	
2011	4:59	3:39	1:20	4:22	4:26	0:04			
Mean	4:25 ± 0:24	3:44 ± 0:07	0:48 ± 0:22	5:20 ± 0:55	4:23 ± 0:35	0:38 ± 0:42	6:47 ± 0:36	6:25 ± 1:35	2:05 ± 0:45

**Table 4:** Race times (h:min ± SD) for the annual three fastest women and men in age groups 75–79 and 80–84 years with GD

Years	75–79 years			80–84 years		
	Women	Men	GD	Women	Men	GD
2004	4:14 ± 0:07	3:46 ± 0:06	0:13 ± 0:08	5:59 ± 1:00	4:08 ± 0:10	0:44 ± 0:19
2005	4:50 ± 0:22	3:52 ± 0:02	0:25 ± 0:08	5:58 ± 0:49	4:05 ± 0:17	0:45 ± 0:10
2006	4:58 ± 0:12	3:49 ± 0:05	0:30 ± 0:03	6:54 ± 0:20	4:27 ± 0:31	0:55 ± 0:12
2007	4:43 ± 0:51	3:58 ± 0:03	0:22 ± 0:15		5:53 ± 0:39	
2008	4:38 ± 0:08	3:58 ± 0:02	0:16 ± 0:02	6:41 ± 0:51	5:05 ± 0:10	0:31 ± 0:12
2009	4:28 ± 0:20	3:34 ± 0:07	0:14 ± 0:06	6:51 ± 0:24	4:47 ± 0:38	0:44 ± 0:12
2010	5:11 ± 0:41	3:46 ± 0:11	0:37 ± 0:13	6:02 ± 1:38	5:04 ± 0:29	0:20 ± 0:18
2011	5:20 ± 0:18	5:58 ± 0:17	0:35 ± 0:03	5:01 ± 1:02	4:41 ± 0:23	0:13 ± 0:04
	4:47 ± 0:21	4:05 ± 0:46	0:24 ± 0:09	6:12 ± 0:40	4:46 ± 0:35	0:36 ± 0:15

## Discussion

This study tested the hypothesis of both an increased participation and an improved performance in elderly marathoners (>75 years) by investigating marathon race times achieved in four of the largest city marathons as part of the ‘World Marathon Majors’ held between 2004 and 2011. The most important findings were, first, participation for female and male runners remained unchanged, second, the fastest women and men became slower and, third, the GD in performance remained unchanged.

### Unchanged participation in female and male participants

A first important finding was that overall participation remained unchanged. However, the number of men increased in the ‘BMW Berlin Marathon’ whereas the number of women and men decreased in the ‘BOA Chicago Marathon’. Generally, an increased in participation in age group marathoners has been observed. For example, Lepers and Cattagni (2012) investigated the changes in participation and performance trends of master marathoners competing between 1980 and 2009 in the ‘New York City Marathon’. They reported that the number of total finishers increased by 65 % between decade 1980–1989 and 1990–1999 and by only 25 % between decade 1990–1999 and 2000–2009. The difference between the present findings and the findings of Lepers and Cattagni (2012) might be explained by the shorter time period in the present study and the inclusion of four different races. Future studies might consider a longer time frame of a single city marathon.

### Decrease in performance in female and male finishers

A second important finding was that marathon race times increased across years for both women and men. This finding disagrees with our hypothesis of an improvement in performance over time. Recent studies investigating age group marathoners reported improved marathon race times in the last ~30–40 years (Jokl et al. 2004; Leyk et al. 2007; Lepers and Cattagni 2012). The most likely reason for these disparate findings could be the short time frame of 8 years and the unchanged participation during these years. Furthermore, we pooled data from four races whereas Jokl et al. (2004) and Lepers and Cattagni (2012) investigated only the ‘New York City Marathon’. Future studies might investigate the performance trends in a large city marathon such as the ‘New York City Marathon’ across a longer period of time than 8 years.

Race times for the fastest women and men in age groups 75–79, 80–84 and 85–89 years were 3:54, 3:34, 4:22, 3:52, 6:06 and 4:37 h:min, respectively. These race times were 0:01, 0:30, 0:10, 0:37, 0:52 and 0:03 h:min slower than the age group world records (Table 5). The age group world records were achieved between 2004 and 2014 and no record was attained at one of the races of the ‘World Marathon Majors’ between 2004 and 2011.

**Table 5:** World records in marathon running for women and men in age groups 75–79, 80–84 and 85–89

Age group	Race time (h:min:s)	Athlete	Age	Years	Race
M 75	3:04:54	Ed Whitlock	76	2007	Rotterdam, NED
M 80	3:15:54	Ed Whitlock	80	2011	Toronto, CAN
M 85	4:34:55	Robert Horman	86	2004	Gold Coast, AUS
W 75	3:53:42	Yoko Nakano	76	2012	Otawara, JPN
W 80	4:12:44	Gwen McFarlan	80	2014	Ottawa, CAB
W 85	5:14:26	Betty Jean McHugh	85	2012	Honolulu, USA

Data from www.world-masters-athletics.org/records

### Unchanged gender difference across years

A third important finding was that the GD in marathon race times remained unchanged across years. The present findings differ from the findings in Lepers and Cattagni (2012) investigating marathon race times of the fastest female and male age group runners aged between 20 and 79 years competing in the ‘New York City Marathon’ between 1980 and 2009. They found that the GD in marathon race times decreased over the last three decades but remained relatively stable across the different age groups during the last decade (Lepers and Cattagni 2012). The GD in marathon race times were significantly lower for decade 2000–2009 compared to both previous decades independent of the age of the athletes (Lepers and Cattagni 2012). The GD in marathon race times were 28.4 ± 10.3, 25.8 ± 6.9, and 19.7 ± 4.2 % for decades 1980–1989, 1990–1999, and 2000–2009, respectively (Lepers and Cattagni 2012). These absolute values were lower compared to our absolute values most likely because they investigated lower age groups (aged between 20 and 79 years). These disparate findings might be the results of the different time periods and the different sample sizes of the studies.

## Conclusions

This study tested the hypothesis that participation would increase and performance would improve in elderly marathoners (>75 years) competing in four of the largest city marathons as part of the ‘World Marathon Majors’ held between 2004 and 2011. Participation for female and male runners remained unchanged considering the four races, the fastest women and men became slower across years and the GD in performance remained unchanged. These findings might be the results of the relatively short period of time of 8 years. Future studies might investigate the performance trends in a large city marathon across a longer period of time.
